# Localization of Oleuropeyl Glucose Esters and a Flavanone to Secretory Cavities of Myrtaceae

**DOI:** 10.1371/journal.pone.0040856

**Published:** 2012-07-20

**Authors:** Allison M. Heskes, Jason Q. D. Goodger, Sammi Tsegay, Tim Quach, Spencer J. Williams, Ian E. Woodrow

**Affiliations:** 1 School of Botany, The University of Melbourne, Victoria, Australia; 2 School of Chemistry and Bio21 Molecular Science and Biotechnology Institute, The University of Melbourne, Victoria, Australia; Instituto de Biología Molecular y Celular de Plantas, Spain

## Abstract

We report the widespread occurrence of structurally diverse oleuropeyl glucose esters, including the new diester eucaglobulin B, localized specifically to the essential oil secretory cavities of myrtaceous species. Clear taxonomic patterns in the composition of cavity extracts within the genus *Eucalyptus* are shown with species from subgenus *Symphyomyrtus* dominated by oleuropeyl glucose esters and species from subgenus *Eucalyptus* dominated instead by the flavanone, pinocembrin. We also examined the intra-species occurrence of oleuropeyl glucose esters by quantifying the abundant constituents cuniloside B and froggattiside A in trees from two populations of *Eucalyptus polybractea* R.T. Baker. All trees contained both compounds, which were positively correlated with total essential oil concentration. This apparent ubiquity of oleuropeyl glucose esters at both intra- and inter-specific levels in *Eucalyptus* is indicative of important physiological or ecological functions. The significance of their prevalence and the sequestration of these esters and also pinocembrin to the extracellular domain of secretory cavities is discussed in light of their potential biological activities and our findings that they are spatially segregated to the exterior of cavity lumina. The localization of oleuropeyl glucose esters to a specific and isolatable tissue type has the potential to aid in future elucidation of function and biosynthesis.

## Introduction

Myrtaceous plants are rich sources of a range of biologically active compounds. In particular, species within the large genus *Eucalyptus* are well known for the abundant essential oils found in their leaves. *Eucalyptus* oil is composed of mono- and sesquiterpenes that possess strong antimicrobial activities [1] and has been implicated in plant defence responses to fungal infection, wounding [2] and folivory [3], [4]. *Eucalyptus* foliage is also characterised by high levels of phenolic compounds including ellagitannins, proanthocyanidins, flavanones, flavonol glycosides, phenolic acids and phloroglucinol derivatives [5], [6], [7]. Many of these are strong antioxidants [8] that may act to quench reactive oxygen species resulting from wounding, pathogen attack and photoinhibition [9], [10]. *Eucalyptus* phenolics can also deter folivory by reducing palatability and the nutritional value of ingested leaves [4], [11].

**Figure 1:**
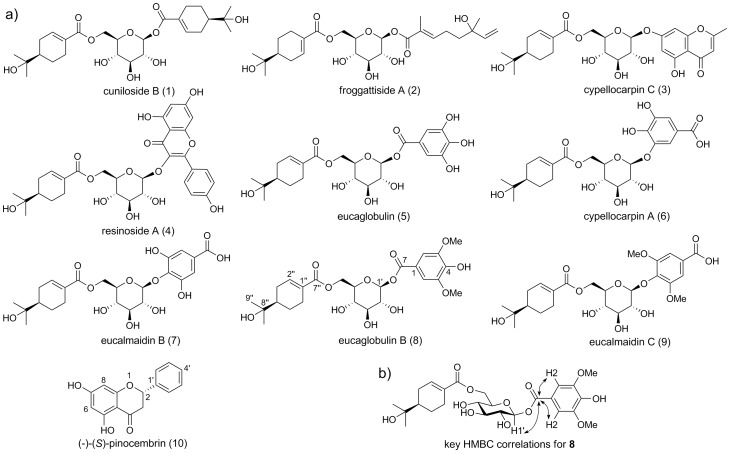
Structures of non-volatile compounds localized to the foliar secretory cavities of *Eucalyptus* species and *Melaleuca armillaris.* (a) Oleuropeyl glucose esters and the flavanone, pinocembrin (**10**) and (b), key HMBC correlations used in the structural elucidation of eucaglobulin B (**8**).

Another group of potentially important biologically active compounds are the monoterpene acid glucose esters. These have been found in a diverse range of plant families but are particularly prevalent in *Eucalyptus* where they have commonly been isolated from bulk leaf extracts, or less often from extracts of fruit capsules (reviewed by [12]). The esters are predominantly composed of the monoterpenoid oleuropeic acid esterified to glucopyranose, generally at the primary hydroxyl position, and either a second monoterpene acid or a phenolic group esterified or glycosylated at the anomeric position (see Fig. 1). They possess an electrophilic α,β-unsaturated carbonyl group in the monoterpenoid and often in the phenolic moiety, and some also have the ability to act as antioxidants through the reducing potential of phenolic hydroxyl groups [12]. Accordingly, some of the *Eucalyptus* esters show significant *in vitro* activity against a range of targets including Epstein-Barr virus [13], *Escherichia coli*, *Candida albicans* and tumor cell lines [14]. These chemical properties combined with their biological activity suggest that this group of compounds may have potential pharmaceutical and therapeutic applications in addition to important physiological and ecological functions.

Unlike the well characterised terpenes and phenolics from *Eucalyptus*, no work has been carried out towards establishing a functional role for oleuropeyl glucose esters *in planta*. Establishing a function is a relatively complex task which is aided by a good understanding of the diversity and ubiquity of the glucose esters in the genus. Some progress has been made in this area. Recently, two of these esters, cuniloside B (**1**) and cypellocarpin C (**3**) were shown to occur in bulk leaf extracts from over 20 species of *Eucalyptus* [15]. In addition, in *E. polybractea*, **1** and froggattiside A (**2**) were found to be exclusively localized to the foliar essential oil secretory cavities [12]. It is not known if secretory cavities are the common site of storage of oleuropeyl glucose esters more generally, but if so it may indicate a possible functional role for the esters within the cavities. Indeed, recent work on enzymatically isolated secretory cavities from *E. polybractea* using multiphoton fluorescence lifetime imaging (MP-FLIM) revealed a unique spatial distribution of the non-volatile component (dominated by **1** and **2**) to the exterior of cavity lumina, abutting the secretory cells [16]. This finding raised the prospect of a functional role of these compounds relating to their localization, possibly as a barrier protecting secretory cells from potentially autotoxic volatile terpenes [16], [17].

The work presented here has three aims, each relating to the function of oleuropeyl glucose esters. The first is to document their ubiquity, abundance and diversity in secretory cavities from a range of *Eucalyptus* species from different sub-genera as well as a species from the sister genus *Melaleuca*. The second aim is to quantify variation in the abundant oleuropeyl glucose esters **1** and **2** in two natural populations of *E. polybractea*, and to investigate their relationship with the essential oils co-housed within secretory cavities. The third aim is to explore the consistency of the recently observed spatial arrangement of the non-volatile fraction within cavity lumina [16] by applying MP-FLIM to isolated secretory cavities from three species of *Eucalyptus*.

## Results and Discussion

### Localization of Oleuropeyl Glucose Esters to the Secretory Cavities of *Eucalyptus*

**Figure 2:**
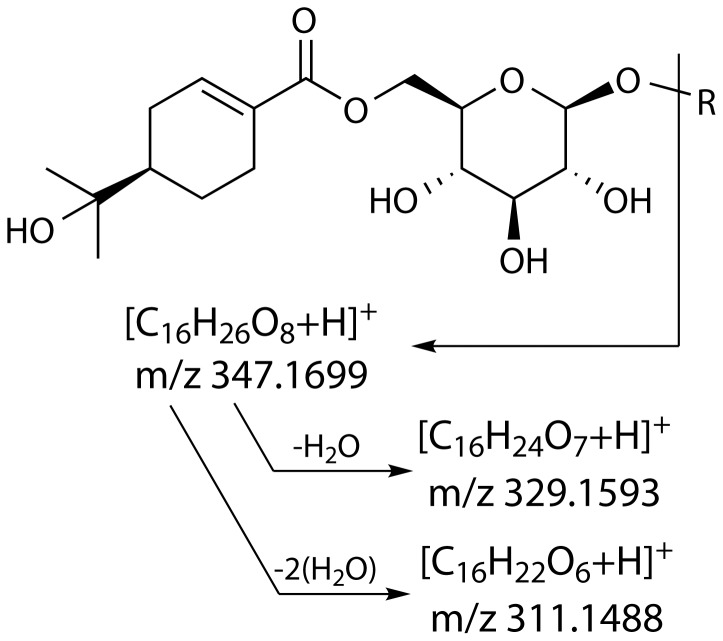
MS^2^ fragmentation of oleuropeyl glucose esters resulting in diagnostic C_16_ fragments.

A selective analysis of the genus *Eucalyptus* was undertaken to examine the occurrence and composition of a non-volatile fraction localised to foliar essential oil secretory cavities. Cavity extracts from 19 species of *Eucalyptus* in three subgenera and a single species of *Melaleuca* were analysed using LC-ESI-FTMS (Table 1). Comparison of MS spectra, UV absorbance and retention times with natural standards showed the secretory cavities of all 19 species of *Eucalyptus* contained **1**, whereas **2** was detected in 15 species and also in *Melaleuca armillaris* (Table 2). In addition, **3** was identified in 15 *Eucalyptus* species based on comparison with a synthetic standard (Table 2). Both **1** and **2** have a distinctive MS^2^ fragmentation pattern with the production of two highly abundant C_16_ fragments of *m/z* 329.1593 and 311.1488, corresponding to an oleuropeic acid esterified to glucose, with the loss of one or two water molecules, respectively ([18]; Fig. 2). A lower abundance C_16_ fragment with *m/z* 347.1699 was also observed, corresponding to an oleuropeyl glucose ester with no loss of water. The fragmentation of **3** resulted in the production of fragment *m/z* 311.1492, also consistent with its structure containing an oleuropeyl glucose ester (Fig. 1a).

**Table 1:** *Eucalyptus* and *Melaleuca* species surveyed for the presence of non-volatile compounds in the lumen of foliar secretory cavities‡.

Species #	Species	Subgenus	Section	Series	Authority
1	*Eucalyptus muelleriana*	*Eucalyptus*	*Capillulus*	*Pachyphloius*	A.W.Howitt
2	*E. gregsoniana*	*Eucalyptus*	*Cineraceae*	*Pauciflorae*	L.A.S.Johnson & Blaxell
3	*E. pauciflora*	*Eucalyptus*	*Cineraceae*	*Pauciflorae*	Sieber ex Spreng.
4	*E. olsenii*	*Eucalyptus*	*Nebulosa*		L.A.S.Johnson & Blaxell
5	*E. erythrocorys*	*Eudesmia*	*Limbatae*	*Heteropterae*	F.Muell.
6	*E. froggattii*	*Symphyomyrtus*	*Adnataria*	*Buxeales*	Blakely
7	*E. dielsii*	*Symphyomyrtus*	*Bisectae*	*Elongatae*	C.A.Gardner
8	*E. platypus*	*Symphyomyrtus*	*Bisectae*	*Erectae*	Hook
9	*E. spathulata*	*Symphyomyrtus*	*Bisectae*	*Erectae*	Hook
10	*E. halophila*	*Symphyomyrtus*	*Bisectae*	*Halophilae*	D.J.Carr & S.G.M.Carr
11	*E. loxophleba* ssp. *lissophloia*	*Symphyomyrtus*	*Bisectae*	*Loxophlebae*	L.A.S.Johnson & K.D.Hill
12	*E. leptophylla*	*Symphyomyrtus*	*Bisectae*	*Porantherae*	F.Muell.
13	*E. myriadena*	*Symphyomyrtus*	*Dumaria*	*Ovulares*	Brooker
14	*E. torquata*	*Symphyomyrtus*	*Dumaria*	*Torquatae*	Luehm.
15	*E. resinifera*	*Symphyomyrtus*	*Latoangulatae*	*Annulares*	Sm.
16	*E. cypellocarpa*	*Symphyomyrtus*	*Maidenaria*	*Globulares*	L.A.S.Johnson
17	*E. globulus*	*Symphyomyrtus*	*Maidenaria*	*Globulares*	Labill.
18	*E. pulverulenta*	*Symphyomyrtus*	*Maidenaria*	*Orbiculares*	Sims
19	*E. dalrympleana*	*Symphyomyrtus*	*Maidenaria*	*Viminales*	Maiden
20	*Melaleuca armillaris*				(Sol. ex Gaertn.) Sm.

‡ *Eucalyptus* taxonomic classification is according to [45].

**Table 2:** Oleuropeyl glucose esters and a flavanone found in secretory cavity extracts of *Eucalyptus* species and *Melaleuca armillaris*.

R_t_ (min)	Observed ESI-LC-FTMS parent ions	Formula	Observed MS^2^ ions of parent ion (bold type)	Compound name	Species # (see Table 1)
5.25	**516.2067 [M+NH_4_]^+^**; 521.1624 [M+Na]^+^	C_23_H_30_O_12_	329.1594 (100); 311.1488 (80); 481.1703 (24);293.1383 (13); 463.1597 (12)	eucaglobulin (**5**),cypellocarpin A (**6**),or eucalmaidin B (**7**)^1^	7,16–19
5.61	**546.2899 [M+NH_4_]^+^**; 551.2456 [M+Na]^+^; 529.2637 [M+H]^+^	C_26_H_40_O_11_	511.2536 (100); 493.2430 (45); 329.1594 (35);347.1700 (21); 311.1490 (19); 475.2321 (14)	unknown 1	6,11,20
6.75	**544.2378 [M+NH_4_]^+^**; 549.1935 [M+Na]^+^	C_25_H_34_O_12_	329.1593 (100); 311.1488 (98); 509.2018 (29)	eucalmaidin C (**9**)	7–9,14
6.77	**546.2899 [M+NH_4_]^+^**; 551.2457 [M+Na]^+^; 529.2637 [M+H]^+^	C_26_H_40_O_11_	511.2535 (100); 329.1594 (59); 493.2430 (29);311.1488 (14); 347.1699 (13)	unknown 2	6,7,11,19,20
7.20	**530.2951 [M+NH_4_]^+^**; 535.2505 [M+Na]^+^; 513.2687 [M+H]^+^	C_26_H_40_O_10_	329.1593 (100); 311.1488 (81); 495.2583 (36);477.2479 (14); 347.1699 (13)	cuniloside B (**1**)^2^	1–20
7.77	**544.2378 [M+NH_4_]^+^**; 549.1935 [M+Na]^+^; 527.2114 [M+H]^+^	C_25_H_34_O_12_	311.1488 (100); 509.2015 (53);491.1909 (39); 329.1595 (21);347.1490 (7); 275.1278 (6);293.1383 (6); 167.1066 (6)	eucaglobulin B (**8**)^2^	7–9
8.00	**615.2061 [M+H]^+^**; 637.1880 [M+Na]^+^	C_31_H_34_O_13_	287.0550 (100); 311.1490 (92)	resinoside A (**4**)	11,16,17
8.06	**521.2009 [M+H]^+^**	C_26_H_32_O_11_	503.1904 (100); 311.1492 (1)	cypellocarpin C (**3**)^2^	2,5–13,15–19
8.23	**505.2635 [M+H]^+^**; 522.2900 [M+NH_4_]^+^; 527.2453 [M+Na]^+^; 487.2529 [M-H_2_O+H]^+^	C_24_H_40_O_11_	193.0495 (100); 311.1488 (86)	unknown 3	5,7–9,13–15
8.39	**530.2950 [M+NH_4_]^+^**; 535.2505 [M+Na]^+^; 513.2685 [M+H]^+^	C_26_H_40_O_10_	329.1593 (100); 311.1488 (71);495.2583 (42); 347.1699 (41);477.2479 (31); 459.2378 (7)	froggattiside A (**2**)^2^	2,3,6–14,16–20
9.27	**602.3884 [M+NH_4_]^+^**607.3439 [M+Na]^+^; 585.3621 [M+H]^+^	C_31_H_52_O_10_	329.1594 (100); 549.3417 (82);311.1487 (32); 347.1699 (21);567.3525 (19);531.3312 (11)	unknown 4	7–9,13,16
9.37	**521.2009 [M+H]^+^**	C_26_H_32_O_11_	503.1904 (100); 485.1809 (2);467.1702 (1); 311.1492 (1)	unknown 5	2–4,7,9,10,12,13,17–19
9.56	**556.2744 [M+NH_4_]^+^**; 561.2296 [M+Na]^+^; 539.2480 [M+H]^+^	C_27_H_38_O_11_	521.2378 (100); 311.1489 (23);539.2488 (14); 503.2272 (11);329.1595 (5)	unknown 6	9,11,17,19
9.66	**522.2900 [M+NH_4_]^+^**; 527.2453 [M+Na]^+^; 505.2635 [M+H]^+^; 487.2530 [M−H_2_O+H]^+^	C_24_H_40_O_11_	487.2535 (100); 469.2431 (43);311.1489 (31); 451.2325 (16);321.1542 (4)	unknown 7	7–9,13
10.79	**602.3885 [M+NH_4_]^+^**; 607.3441 [M+Na]^+^; 585.3625 [M+H]^+^	C_31_H_52_O_10_	549.3419 (100); 531.3314 (95);329.1594 (77); 567.3527 (58);311.1489 (35); 347.1698 (9)	unknown 8	7–9
13.27	**553.2635 [M+H]^+^**	C_28_H_40_O_11_	535.2537 (100); 517.2427 (28);311.1487 (24)	unknown 9	7–9,11,16,17,19
17.06	**255.0661 [M−H]^−^**; 511.1386 [2 M−H]^-^	C_15_H_12_O_4_	213.0551 (100); 151.0032 (97);187.0761 (48); 211.0760 (35);169.0655 (30); 183.0813 (21)	pinocembrin (**10**)^2^	1–4

1 Spectral data consistent with all three compounds.

2 Identification by comparison with authentic standards or NMR analyses.

We used the characteristic fragmentation pattern of the oleuropeyl glucose ester moiety as a means to search for other structurally related compounds in the non-volatile extracts of secretory cavities. The resultant mass spectra were consistent with eight oleuropeyl glucose esters previously reported from bulk leaf extracts of *Eucalyptus* (Table 2, Fig. 1a). For example, a pseudomolecular ion peak [M+H]^+^ at *m/z* 615.2061 and [M+Na]^+^ at *m/z* 637.1880, corresponding to the molecular formula C_31_H_34_O_13_, matched published data for resinoside A (**4**; [19]). Furthermore, fragmentation of the [M+H]^+^ ion resulted in the fragments *m/z* 287.0550 and 311.1490, consistent with the presence of a kaempferol moiety and an oleuropeyl glucose ester, respectively. Interestingly, no mass spectral data matching **4** was detected in the extract from *E. resinifera* secretory cavities, the species from which this compound was first isolated from whole leaf extracts; however, a spectral match was detected in the extract of *E. cypellocarpa* cavities, a species from which **4** has been previously reported in leaves [20]. The mass spectrum of a compound eluting at 5.25 min in five of the species matched three structurally related compounds previously reported from *Eucalyptus* leaf extracts: eucaglobulin (**5**) [21], cypellocarpin A (**6**) [13] and eucalmaidin B (**7**) [22]. Further analyses are required to differentiate between isomers that have gallic acid attached in different ways to the anomeric position of glucose (Fig. 1a).

The cavity extracts of the majority of species also contained up to nine unidentified oleuropeyl glucose ester containing compounds (Table 2). The subgenus *Symphyomyrtus* was found to be particularly rich in these putative esters with *E. dielsii,* and *E. spathulata* containing eight and *E. platypus* containing six of these compounds. Many of the unknown esters were found in extracts of multiple species. One such compound was present in three species and was particularly abundant in the cavity extract from *E. platypus*. The compound was purified from that species, subjected to MS and NMR analyses and identified as a new compound, named here as eucaglobulin B (**8**), owing to its close structural relationship to eucaglobulin (**5**).

The molecular formula of **8** was established by HR-ESI-FTMS (*m/z* 544.2378 [M+NH_4_]^+^; 549.1935 [M+Na]^+^; 527.2114 [M+H]^+^) as C_25_H_34_O_12_. Fragmentation of the [M+NH_4_]^+^ ion resulted in the characteristic 311, 329 and 347 daughter ions, consistent with the presence of an oleuropeyl glucose ester. Fragmentation of the [M−H]^−^ ion gave a daughter ion of *m*/*z* 197.0448 consistent with a dimethylgallic acid group. The presence of a dimethylgallyl group and an oleuropeyl group suggested structural similarity or identity with the known compound eucalmaidin C (**9**). The ^1^H NMR spectrum appeared almost identical to that reported for **9**, supporting the presence of oleuropeyl and 3,5-dimethylgallyl esters, with the exception of the anomeric proton being present at δ 5.67 ppm (rather than δ 4.88 as reported for **9**). In particular, the downfield shifts of both H1′ and H6′a and H6′b are consistent with ester linkages at these positions. HMBC spectroscopy revealed correlations between H2 and C7 of the 3,5-dimethylgallyl fragment, and between C7 of the 3,5-dimethylgallyl fragment and the glucopyranose H1′, confirming that the 3,5-dimethylgallic acid group was attached to the anomeric position of the glucose (C1′), and the oleuropeic acid group at C6′ (Fig. 1b). Based on these analyses, the structure of **8** was proposed.

It appears the occurrence of oleuropeyl glucose esters is widespread in eucalypts. Along with the evidence presented here, compounds **1** and **3** were recently found in bulk leaf extracts of a further 10 species from three *Eucalyptus* subgenera and the sister genus *Corymbia* [15]. Moreover, it appears that most, if not all of these compounds are localised to the lumen of foliar secretory cavities. Furthermore, the positive identification of **1** and **2** as well as the identification of two other putative esters in a species from the genus *Melaleuca* suggests that non-volatiles localized to secretory cavities may be more widespread in the family Myrtaceae. Interestingly, all species from other families in which oleuropeyl glucose esters have been isolated from bulk leaf extracts produce essential oils and, with the exception of *Olea europea*, all produce and store essential oils in specialised storage structures [23], [24], [25]. It is tempting to speculate that the esters may also be housed alongside essential oils in specialised secretory structures in the non-myrtaceous species.

The diversity, ubiquity and abundance of oleuropeyl glucose esters across many *Eucalyptus* species are suggestive of important ecological or physiological roles. A common structural theme shared among these esters is the presence of at least one α,β-unsaturated carbonyl group in the monoterpene acid moieties and often another in the phenolic moiety, if present. This functional group has been shown to be an important determinant of biological activity in a range of molecules including those specifically involved in plant defence [26], [27]. The reactivity of the α,β-unsaturated carbonyls in combination with the reducing potential of any phenolic hydroxyls suggests sequestration to extracellular domains may be a mechanism to avoid potential auto-toxicity within leaves, similar to that proposed for the sequestration of mono- and sesquiterpenes to specialized secretory structures [28].

Based on the LC-PDA and MS-TIC data, the non-volatile extracted from secretory cavities of four species belonging to the subgenus *Eucalyptus* (*E. meulleriana, E. pauciflora, E. gregsoniana and E. olsenii*) were dominated by a compound that did not show the characteristic MS^2^ fragmentation of an oleuropeyl glucose ester, despite such esters being present at lower levels. Instead, the dominant compound had a pseudomolecular ion peak [M-H]^−^ at *m/z* 255.0661, corresponding to the molecular formula C_15_H_12_O_4_. ^1^H and ^13^C NMR analysis and optical rotation identified this compound as (−)-(*S*)-pinocembrin (**10**), a flavanone found in a diverse array of species. Of special relevance is its identification in the resinous exudates of leaf glands (including glandular trichomes) of several species including *Populus deltoides* (Salicaceae) [29], *Lychnophora ericoides* (Asteraceae) [30], *Adenostoma sparsifolium* (Rosaceae) [31] and *Acacia neovernicosa* (Fabaceae) [32]. There is only a single report of its isolation from the leaves of a eucalypt (*E. sieberi*, also from subgenus *Eucalyptus* [33]), although it has been reported as a constituent of unifloral honey derived from several eucalypt species [34]. Its localization to the surface secretions of plants is suggestive of a role in defence and indeed it has been shown to be an effective antibacterial, antifungal and antifeedant agent [29], [35], [36]. These activities may relate to the presence of the phenolic groups in its structure (Fig. 2a). Once again, it is possible that sequestration of **10** to secretory cavities is related to its potential for auto-toxicity to the plant.

### Intraspecific Variation of 1, 2 and Total Essential Oils

**Figure 3:**
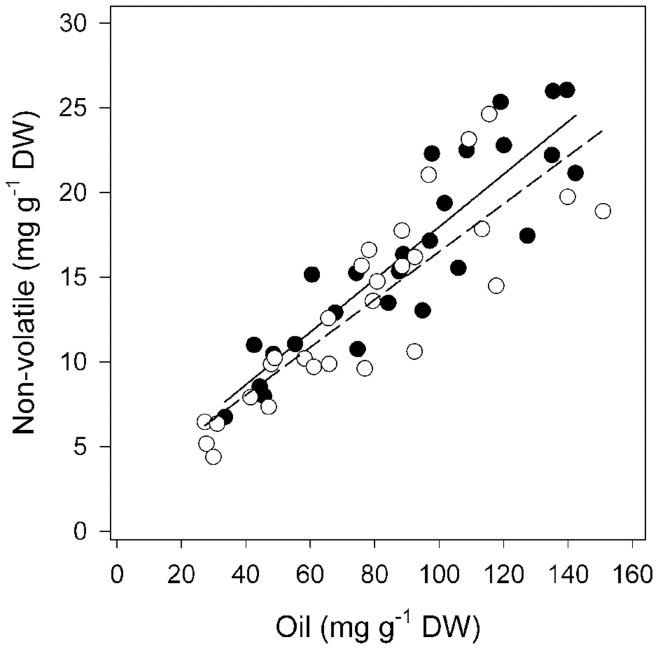
Relationship between total oil (mg g^−1^ DW) and non-volatiles (combined cuniloside B (1) and froggattiside A (2); mg g^−1^ DW) for two populations of *E. polybractea* (closed circles population A; open circles population B). Linear regressions were significant with equations of *non-volatiles* = 2.44+0.15×*total oil* for Population A (solid line; *r* ^2^ = 0.80; F = 96.23, *P* = 0.00) and *non-volatile*  = 2.40+0.14×*total oil* for Population B (dashed line; *r* ^2^ = 0.73; F = 70.99, *P* = 0.00).

Leaf samples from two *E. polybractea* populations were analysed for foliar non-volatiles (based on HPLC quantification of **1** and **2** only) and total essential oil content (GC-FID). Mean (± SE) total oil in population A was 89.7±6.5 mg g^−1^ dry weight (DW) and in population B was 76.7±6.2 mg g^−1^ DW. The GC profile of all trees was dominated by 1,8-cineole with respective mean percentage abundances (±SE) of 78.5±0.8 and 71.9±1.4. All trees tested contained both **1** and **2** with mean values (± SE) of 14.8±1.0 and 1.5±0.1 mg g^−1^ DW, respectively for population A and 11.6±0.9 and 1.6±0.2 mg g^−1^ DW, respectively for population B. Strong positive correlations were found between the concentration of **1** and **2** and the concentration of oil on a dry leaf basis in both populations (*r* ^2^ = 0.80 and 0.73 in populations A and B, respectively; Fig. 3).

The strong positive correlations could arise from various factors. First, the components that make up the non-volatile and oil may be biosynthetically linked. Secondly, the non-volatiles and oil may be responding to related selection pressures. Thirdly, the non-volatiles may play a functional role in oil storage. A similarly positive correlation was found in multiple *Eucalyptus* species between the monoterpene 1,8-cineole and sideroxylonal, a non-volatile formylated phloroglucinol compound (FPC) [37]. FPCs are not known to be stored in secretory cavities and are thought not to be biosynthetically related to monoterpenes, but they were found to map to the same quantitative trait loci in *E. nitens* [38]. It was suggested that a common regulatory region controlling gene transcription may be responsible for the observed correlations [38]. Nonetheless, a link between sideroxylonal and terpene concentration and possum feeding choice can also explain the positive correlations in evolutionary terms due to the demonstrated role of volatile terpenes in creating a conditioned flavour aversion in marsupial folivores [39]. A similar interaction may be occurring between the non-volatile oleuropeyl esters and terpenes in *Eucalyptus*, although in this case, the strong likelihood of a shared biosynthetic pathway must be taken into consideration due to the presence of the monoterpene-derived terpenoid moieties.

### MP-FLIM of in situ Secretory Cavity Components

**Figure 4:**
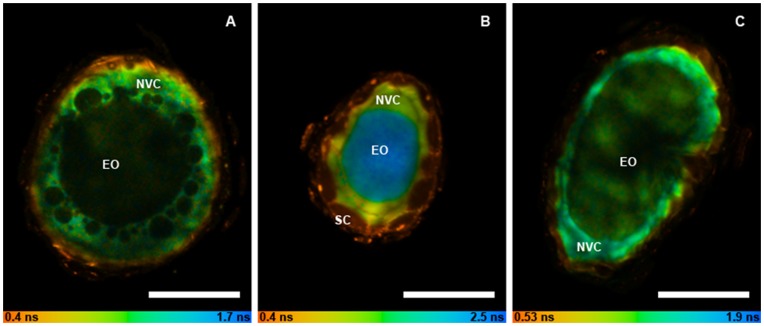
Fluorescence lifetime images of enzymatically isolated eucalypt secretory cavities showing localization of the lumen non-volatile component. Eucalypt species: *E. polybractea* (A), *E. froggattii* (B) and *E. spathulata* (C). Pseudocolour mapping represents mean fluorescence lifetime. Secretory cells (SC); non-volatile component (NVC); essential oil (EO). Scale bar represents 50 µm.

We examined the localization of autofluorescent constituents of the non-volatile component within isolated secretory cavities from *E. spathulata*, and *E. froggattii* to see if the non-volatile and oil were spatially segregated as recently reported for *E. polybractea* [16]. The non-volatile component in cavities from *E. spathulata* and *E. froggattii* was found to be highly autofluorescent and images of the cavity lumina clearly show it abutting the secretory cells in a comparable manner to *E. polybractea,* albeit with markedly different fluorescence lifetimes (as indicated by the pseudo-colour mapping; Fig. 4). Purified compound **8**, identified in *E. spathulata*, was analysed for its fluorescent properties. It was found to be autofluorescent, with respective excitation and emission maxima of 275 nm and 343 nm (consistent with the presence of a gallic acid moiety), and capable of multiphoton excitation. Hence it is likely **8** is contributing to the fluorescence of the non-volatile component in the isolated cavities from this species. In addition, the gallic acid glycoside **9** was detected in *E. spathulata* and may also be contributing to the observed fluorescence. The major non-volatile constituents from *E. polybractea* and *E. froggattii* cavities (compounds **1** and **2**) are non-fluorescent and no evidence was found for the presence of esters with gallic acid moieties in the extract of *E. froggattii*; however, there is evidence for other phenolic esters which are likely responsible for the observed fluorescence (Table 2).

The occurrence of non-volatile components in essential oil secretory structures has been noted in other species and a protective function was proposed for secretions observed in the subcuticular space of mint trichomes [40]. The common localization of these components to the exterior of cavity lumina supports their speculated function as a region of low diffusivity between the secretory cells and essential oil that may act to protect the cells from the potential autotoxic effects of the oil [12], [16]. Nonetheless, as noted above, sequestration of such potentially biologically active metabolites to extracellular secretory cavities may simply be a means of avoiding auto-toxicity from the non-volatiles themselves. If so, their spatial arrangement within cavity lumina may merely be a result of their relative hydrophilicity compared to terpenes, resulting in secretion into and accretion around the periphery of the lumina, rather than any specific functional role related to this arrangement. More research is required to probe the relationship between the non-volatile component and essential oils co-housed within secretory cavities.

## Conclusion

Oleuropeyl glucose esters were identified as common components of secretory cavity extracts in all *Eucalyptus* species examined. Particular species within the subgenus *Symphyomyrtus* proved to be rich in these esters and would make ideal targets for future biosynthetic studies or could act as sources of these interesting natural products. The common distribution of these compounds within the genus *Eucalyptus* and potentially more broadly within the Myrtaceae, as well as their commonality at an intra-specific level and tight correlation with essential oils, is indicative of important functional roles perhaps in plant defence. Moreover, the localisation of these biologically active compounds to secretory cavity lumina provides a strong indication that they may be sequestered there to avoid possible autotoxic effects. The localization of non-volatile constituents to secretory cavities is of particular interest given the recent development of a protocol to isolate live *Eucalyptus* secretory cavities from leaves [17], which may be used to help elucidate their biosynthetic pathways and function *in planta*.

## Materials and Methods

### Ethics Statement

All necessary permits were obtained for the field studies as described below.

### Plant Material

Species were selected based on published descriptions of large foliar secretory cavities and essential oil yield and also to represent different subgenera of the genus *Eucalyptus*. Bulk samples of fully expanded leaves were collected in April 2009 from *Eucalyptus* trees growing in the Peter Francis Points Arboretum (Colerain, Australia, 37°36.57′S, 141°41.05′E; permission for sample collection was gained from the arboretum curator Ray Clay) and stored at −80°C until analysis. Leaves of *Melaleuca armillaris* were collected and analysed immediately from trees growing at the University of Melbourne, Parkville, Victoria.

### *Eucalyptus* Polybractea Population Survey

Bulk samples of fully expanded leaves were collected in February 2011 from two natural populations of *E. polybractea* from north-western Victoria, Australia (population A: 36°31.87′S, 143°44.68′E, population B: 36°32.78′S, 143°44.93′E) located on public land (Parks Victoria collection permit: 10004785) subjected to short-rotation coppicing approximately 2 km apart (see [41]). At the time of harvesting the coppice was 18 months of age. Samples were collected along a 30 m north-south transect with only fully expanded leaves from the current seasons growth harvested. Leaves were kept on ice for no longer than 5 h before being transferred to a −80°C freezer. Duplicate leaves for each tree were ground to a fine powder under liquid nitrogen in a mortar and pestle, extracted in 3 mL hexane containing 100 µg mL^−1^ tridecane as an internal standard and incubated at 50°C for 4 days. Samples were analysed by GC-FID following the protocol of [42]. Tissue extracted for oil was allowed to dry and then re-extracted with 3 mL 70% acetone at 25°C for 24 h. 180 µL aliquots of acetone extract were made up to 1.20 mL with deionized H_2_O and loaded onto 30 mg Strata-X reverse phase cartridges for solid-phase extraction (Phenomenex, Torrance, USA,) preconditioned with 100% methanol followed by H_2_O. Samples were washed with 1 mL 30% methanol in water and then **1** and **2** were eluted with 3 mL 80% methanol. This fraction was further fractionated by RP-HPLC using the Shimadzu HPLC system under the following conditions: Gemini C18 analytical column (Phenomenex, 5 µm, 150×4.6 mm); flow rate, 1 mL min^−1^; column temperature, 23°C; gradient (acetonitrile/water), 30–45% over 2 min followed by 45–65% over 8 min. Both **1** and **2** were quantified based on PDA responses at 220 nm compared to a standard series of synthetic **1** [15].

### Collection of Non-volatile Fraction from Secretory Cavities

With the aid of a stereomicroscope, individual leaves from each species were cut into ∼2 mm wide strips, held under water with forceps and the non-volatile contents of secretory cavities physically removed from the cut edges of the leaf strips using a microprobe with a 1 µm tip (World Precision Instruments, Sarasota, USA). The microprobe tip was then rinsed in a vial of acetonitrile (100%) to dissolve the collected non-volatile material. This process was continued until sufficient non-volatile was collected for analysis. Each acetonitrile collection was dried under a constant stream of N_2_, re-dissolved in acetonitrile (70%), passed through a 0.45 µm filter and analysed using the LC-MS system.

### LC-MS Analyses

The LC-MS system used for fractionation and accurate mass measurements was comprised of a Finnigan Surveyor LC Pump, Surveyor AutoSampler and a linear ion trap coupled to a FT-ICR mass spectrometer LTQ FTMS (Finnigan MAT, Bremen, Germany). The instrument was calibrated weekly with Agilent G2421A solution (Agilent Technologies, Santa Clara, USA) for positive and negative ion mode. The following chromatographic conditions were used for the separation of secretory cavity extracts: Gemini C18 analytical column (Phenomenex, 5 µm, 150×4.6 mm); flow rate, 0.5 mL min^−1^; column temperature, 23°C; gradient, 20–35% acetonitrile/water acidified with acetic acid (0.1%) over 2 min, followed by 35–65% over 14 min, then 65–100% over 3 min. The following MS source conditions were used in positive ion mode: sheath gas, 60 arbitrary units; spray voltage, 5.3 kV; capillary temperature, 250°C; capillary voltage, 33 V; and tube lens voltage, 80 V. In negative ion mode: sheath gas, 60 arbitrary units; spray voltage, 2.7 kV; capillary temperature, 250°C; capillary voltage, −12 V; and tube lens voltage, −140 V. In both positive and negative mode a scan range of 200–1000 *m*/z was used and CID carried out at 35% normalized collision energy. Mass spectra were analysed with Xcalibur software (Thermo Electron, San Jose, USA). Identification of **1** and **2** was based on spectral similarity, UV absorbance and retention time of natural standards purified from *E. froggattii* [18]. Identification of **3** was based on spectral similarity and retention time of a synthetic standard [15].

### IR and NMR Spectroscopy

NMR spectra were recorded on Varian 500, Bruker AV 600 (600, 150 MHz), or Bruker Biospin-Avance 800. Chemical shifts (δ) for ^1^H NMR spectra are reported in parts per million and are followed by multiplicity, coupling constant(s) (*J*, Hz), integration and assignments. The following abbreviations are used in reporting multiplicities: s, singlet; d, doublet; ABq, AB quartet; br, broad. Residual solvent signals were used for reference: δ 7.26 for ^1^H NMR in CHCl_3_, δ 2.05 for ^1^H NMR and δ 29.84 for ^13^C NMR in *d*_6_-acetone. IR spectra were obtained on a Perkin–Elmer Spectrum One FTIR spectrometer with a zinc selenide/diamond Universal ATR sampling accessory as a thin film.

### Structural Elucidation of Eucaglobulin B

Eucaglobulin B (**8**) was purified for structure elucidation from bulk leaf extracts of *E. platypus.* Leaf samples were ground under liquid N_2_ to a fine powder and extracted in 70% acetone for 24 h at 25°C. The extract was loaded onto a 200 mg Strata-X reverse phase cartridge for solid-phase extraction (Phenomenex) and eluted successively with 10, 20 and 30% acetonitrile. The 20% fraction was dried under a stream of N_2_ and then redissolved in 50% acetonitrile acidified with 0.1% formic acid. Purification of **8** from this fraction was carried out on a HPLC system comprised of a Shimadzu LC-20AT pump, SIL-20A HT autosampler, SPD-M20A detector and FRC-10A fraction collector (Shimadzu Corporation, Kyoto, Japan) under the following conditions: Gemini C18 analytical column (Phenomenex, 5 µm, 150×4.6 mm); flow rate, 1 mL min^−1^; column temperature, 23°C; gradient, 20–36% acidified acetonitrile (0.1% formic acid) over 15 min.

[α]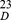 +8.9 (*c* 0.04, CHCl_3_); IR ν 3380, 2937, 1703, 1599, 1462, 1426, 1337, 1257, 1216, 1185, 1070, 1035, 762 cm^−1^; ^1^H NMR (600 MHz, *d*_4_-MeOH) δ 7.41 (s, 2 H, ArH), 7.03-7.02 (m, 1H, H2′′), 5.68-5.67 (m, 1H, H1′), 4.45 (dd, *J* = 12.0, 1.8 Hz, 1H, H6′), 4.27 (dd, *J* = 12.0, 5.4 Hz, 1H, H6′), 3.90 (s, 6H, OMe), 3.70-3.67 (m, 1H, H5′), 3.54-3.49 (m, 2H, H2′,3′), 3.46-3.42 (m, 1H, H4′), 3.33 (m, 1H, H2′), 2.51-2.47 (m, 1H, H6′′), 2.39-2.31 (m, 1H, H3′′), 2.17-2.10 (m, 1H, H6′′), 2.04-1.98 (m, 2H, H5′′/H3′′), 1.56-1.51 (m, 1H, H4′′), 1.20 (dd, *J* = 12.6, 4.8 Hz, 1H, H5′′), 1.17 (2 s, 2×3H, H9′′,H10′′); ^13^C NMR (200 MHz, d_4_-methanol) δ 167.33 (C7′′), 165.13 (C7), 147.37 (C3), 141.18 (C4), 129.59 (C1′′), 118.87 (C1), 107.18 (C2), 104.75 (C2′′), 94.55 (C1′), 44.13 (C6′′), 27.13 (C5′′), 25.34 (C4′′) (Note: Owing to the small amount of material available, the reported ^13^C data was obtained from HMBC spectrum. Non-ambiguous data could not be obtained for the following carbons: C3′′, C8′′, C9′′, C2′, C3′, C4′, C5′, C6′.). HRMS (ESI^+^) *m*/*z*: 544.2378 [M+NH_4_]^+^ (calcd. 544.2399 for C_25_H_38_O_12_NH_4_) and *m*/*z* 549.1935 [M+Na]^+^ (calcd. 549.1953 for C_25_H_34_O_12_Na). MS^2^ fragmentation of [M+NH_4_]^+^: 311.1488 (100); 509.2015 (53); 491.1909 (39); 329.1595 (21); 347.1490 (7); 275.1278 (6); 293.1383 (6); 167.1066 (6).

### Structural Elucidation of (–)-(S)-pinocembrin

Pinocembrin (**10**) was purified for structure elucidation from secretory cavity extracts of *E. olsenii.* Purification of **10** was carried out on the Shimadzu LC system under the following conditions: Gemini C18 analytical column (Phenomenex, 5 µm, 150×4.6 mm); flow rate, 1 mL min^−1^; column temperature, 23°C; gradient, 65–100% acetonitrile/water over 5 min.

[α]_D_^22^ −64.6° (c 0.025 in acetone). Lit. [33] −45.3 (c 0.9 in acetone, 15°C); lit. [43] −45.6° (c 0.5 in MeOH);^ 1^H NMR (CDCl_3_, 500 MHz) δ 12.04 (s, 1H, 5-OH), 7.47-7.38 (m, 5H, Ar), 6.00 (ABq, *J* = 2.5 Hz, 2H, H6 and H8), 5.43 (dd, *J* = 12.5, 3.0 Hz, 1H, H2), 3.09 (dd, *J* = 17.0, 13.0 Hz, 1H, H3b), 2.83 (dd, *J* = 17.0, 3.0 Hz, 1H, H3b). This data is consistent with that reported [43]; ^1^H NMR (*d*_6_-acetone, 500 MHz) δ 12.19 (br s, 1H, 5-OH), 7.57-7.55 (m, 3H, Ar), 7.46-7.38 (m, 2H, Ar), 5.96 (d, *J* = 2.0 Hz, 1H, H8), 5.92 (d, *J* = 2.0 Hz, 1H, H6), 5.54 (dd, *J* = 12.5, 3.2 Hz, 1H, H2), 3.12 (dd, *J* = 17.0, 12.5 Hz, 1H, H3a), 2.77 (dd, *J* = 17.0, 3.2 Hz, 1H, H3b); ^13^C NMR (*d*_6_-acetone, 150 MHz) δ 140.0 (C1’), 129.4 (C3’), 129.2 (C4’), 127.2 (C2’), 97.4 (C6), 96.5 (C8), 79.7 (C2), 43.6 (C3) (Note: No signals were observed for the quaternary carbons in the ^13^C NMR spectrum due to the low concentration of the sample; however, the non-quaternary signals in the ^13^C NMR spectrum and all the signals in the ^1^H NMR spectrum were consistent with literature values [44]). HR-FTMS (ESI^−^) *m*/*z*: 255.0661 [M-H]^−^ (calcd. 255.0663 for C_15_H_11_O_4_). MS^2^ fragmentation of [M-H]^−^: 213.0551 (100); 151.0032 (97); 187.0761 (48); 211.0760 (35); 169.0655 (30); 183.0813 (21).

### MP-FLIM Imaging and Fluorescence Spectrometry

Secretory cavities were isolated from leaf tissue using a pectinase leaf digestion protocol as described in [17]. However, the digestion protocol was altered for *E. spathulata* from 12 h to 2 h. Isolated cavities were imaged and analysed as reported in [16]. Purified **8** was dissolved in 100% acetonitrile and excitation and emission spectra collected using a Cary Eclipse fluorescence spectrophotometer (Varian Inc, Palo Alto, CA, USA) with a spectral bandwidth of 5 nm. To test for the ability of **8** to undergo multiphoton excitation the compound was dried onto a coverslip and imaged as per the cavities.
